# The challenges of including sex/gender analysis in systematic reviews: a qualitative survey

**DOI:** 10.1186/2046-4053-3-33

**Published:** 2014-04-10

**Authors:** Vivien Runnels, Sari Tudiver, Marion Doull, Madeline Boscoe

**Affiliations:** 1 Globalization and Health Equity Research Unit, Institute of Population Health, University of Ottawa, 1 Stewart Street, Ottawa, ON K1N 6N5, Canada; 2 Gender and Health Consultant/Researcher, 161 Northwestern Ave, Ottawa, ON K1Y 0M1, Canada; 3 School of Population and Public Health, Faculty of Medicine, University of British Columbia, 414-2206 East Mall, Vancouver, BC V6T 1Z3, Canada; 4 Reach Community Health Centre, 1145 Commercial Drive, Vancouver, BC V5L 3X3, Canada

**Keywords:** Gender, Qualitative methods, Sex, Sex/gender analysis, Systematic review methods

## Abstract

**Background:**

Systematic review methodology includes the rigorous collection, selection, and evaluation of data in order to synthesize the best available evidence for health practice, health technology assessments, and health policy. Despite evidence that sex and gender matter to health outcomes, data and analysis related to sex and gender are frequently absent in systematic reviews, raising concerns about the quality and applicability of reviews. Few studies have focused on challenges to implementing sex/gender analysis within systematic reviews.

**Methods:**

A multidisciplinary group of systematic reviewers, methodologists, biomedical and social science researchers, health practitioners, and other health sector professionals completed an open-ended survey prior to a two-day workshop focused on sex/gender, equity, and bias in systematic reviews. Respondents were asked to identify challenging or ‘thorny’ issues associated with integrating sex and gender in systematic reviews and indicate how they address these in their work. Data were analysed using interpretive description. A summary of the findings was presented and discussed with workshop participants.

**Results:**

Respondents identified conceptual challenges, such as defining sex and gender, methodological challenges in measuring and analysing sex and gender, challenges related to availability of data and data quality, and practical and policy challenges. No respondents discussed how they addressed these challenges, but all proposed ways to address sex/gender analysis in the future.

**Conclusions:**

Respondents identified a wide range of interrelated challenges to implementing sex/gender considerations within systematic reviews. To our knowledge, this paper is the first to identify these challenges from the perspectives of those conducting and using systematic reviews. A framework and methods to integrate sex/gender analysis in systematic reviews are in the early stages of development. A number of priority items and collaborative initiatives to guide systematic reviewers in sex/gender analysis are provided, based on the survey results and subsequent workshop discussions. An emerging ‘community of practice’ is committed to enhancing the quality and applicability of systematic reviews by integrating considerations of sex/gender into the review process, with the goals of improving health outcomes and ensuring health equity for all persons.

## Background

Sound decisions about clinical interventions and about implementation of health policies ideally require a strong evidence base combined with judgment about how the evidence could best be applied to a particular patient and/or in a particular policy context. Systematic reviews provide a framework, methods, and tools to collate and analyse relevant evidence from primary research. They are widely used to guide clinical, policy and program decisions, conduct health technology assessments, and determine directions for new research [1-4]. They are also used to assess how interventions may contribute to health equity [5]. However, many systematic reviews do not clearly address the applicability of the evidence [6]. Of particular interest here, most do not explicitly describe whether the evidence applies to both women and men, nor analyse possible sex and gender-related differences and similarities, nor indicate when such data are missing [7,8].

Sex refers to the biological, genetic, and physiological processes that generally distinguish females from males while gender refers to the roles, relationships, behaviours, relative power, and other traits that societies generally ascribe to women, men, and people of diverse gender identities (e.g., transgender persons) [9,10]. Although often categorized as binary for analysis (e.g., male/female; masculine/feminine), processes of sex/gender are dynamic, multidimensional, and interactive [11-13]. Sex and gender analysis, referred to here as sex/gender analysis to emphasize the interrelationships among the concepts, is an analytical framework that is used to explore possible biological and social similarities and differences between and among men and women, boys and girls [9]. For example, in the context of health care systems, sex/gender analysis explores the interrelationships of sex and gender within or between groups in order to identify how these may affect health experiences, access to care, and health outcomes.

The lack of attention to sex/gender in systematic reviews is of concern for several reasons. There is a substantive and growing body of knowledge calling attention to sex/gender-related differences in pharmacokinetics and pharmacodynamics, genetic expression, and in prevalence, onset, and severity of diseases such as auto-immune disorders [14-21]. Research studies also identify sex/gender differences in subjective experiences of conditions such as depression and chronic pain, responses to health interventions, the utilization of health care systems, and in many other processes that influence health [22-25]. Failure to consider whether such differences are clinically meaningful, and how processes related to sex/gender may or may not be relevant to a particular intervention, can result in inappropriate application of findings and have harmful implications for clinical outcomes and/or for the success of health policies and programs [26,27].

Systematic reviews can also be used to help determine which interventions reduce inequities in health, defined as preventable and therefore unfair differences in health between groups [28-31]. Explicitly identifying to whom the evidence does or does not apply is necessary to formulate social policy initiatives to reduce poverty and other inequities and to determine what interventions are appropriate with particular populations. It is widely recognized that sex/gender-related processes are determinants of health – men, women, and persons of diverse gender identities may experience disadvantages and marginalization in different ways, both within and between communities [32-35]. Further, many forms of bias, including publication bias (the tendency to publish positive rather than negative research findings); and outcome reporting bias (the selective reporting of some outcomes, but not others, depending on the nature and direction of the results), are a major concern for systematic review methods and can affect applicability and quality of studies [36]. Lack of reporting about sex/gender in systematic reviews can elevate risk of bias and limit reviewers, researchers and other users of reviews from assessing in what ways, and specifically for whom, interventions may or may not be effective and may reduce or exacerbate health inequities.

Furthermore, many research and governmental organizations have policies mandating that sex/gender-related issues be addressed in proposals for health research funding, clinical trial design and registration, and in policy and program initiatives, citing the rationale that consideration of possible sex/gender-related similarities and differences is essential for good science, policies, and programs [37-39]. Editors of a number of major health journals are beginning to call for sex/gender policies to enhance the quality and applicability of reporting of research evidence [40-43].

Integrating sex/gender analysis into the processes and methods of systematic reviews can enhance the applicability and quality of reviews, with the long term goals of improving health outcomes and reducing health inequities. While many review authors, editors, and group coordinators acknowledge there is value to considering sex/gender, they point to challenges in implementing such analysis. To address these concerns, our Working Group on Sex and Gender Analysis in Systematic Reviews^a^ convened a meeting of review authors, review group coordinators and editors, methodologists, health practitioners, biomedical and social science researchers, consumers, and funders to discuss the factors that hinder the integration of sex/gender analysis within systematic reviews and to outline priorities for addressing these challenges. Invited participants were individuals we knew or were known to us through research, academic, and policy networks as possessing knowledge, practical experience, and expertise in relation to systematic reviews and/or in the application of sex/gender analysis to health evidence. To our knowledge, this paper is the first to identify the challenges of integrating sex/gender analysis in systematic reviews from the perspectives of those conducting and using systematic reviews.

## Methods

A brief, open-ended survey was distributed by email two weeks prior to a two-day workshop focused on sex/gender, health equity, and bias in systematic reviews.^b^ Those invited were asked to briefly describe their backgrounds, how they came to be interested in systematic reviews and quality in health research, and what outcomes they would like to see from the meeting. The central questions asked were: “*What do you perceive to be the challenges of incorporating sex/gender, equity, and bias in systematic reviews? What are some of the most perplexing, stimulating or ‘thorny’ questions/issues that have emerged in your work with systematic reviews?* and *How have you attempted to address these?*” [Respondents could select challenges and/or ‘thorny’ issues to address the latter question]. The main goals of the survey were to provide organizers with a better understanding of the interests of workshop participants in systematic reviews and/or in sex/gender-related issues, to identify a diverse range of challenges beyond what we might have considered, and to encourage respondents to think about challenges and possible solutions to better inform workshop discussions.

### Analysis

All responses to the questions were coded systematically by one analyst (VR) using qualitative data analysis software to organize the coded responses.^c^ Units of coding were determined as having discrete meaning as the analyst interpreted them [44-46]. The data were analyzed in accordance with qualitative description, a low-inference interpretive approach to qualitative data analysis, and designed specifically for the purpose of description [44,47]. Each unit was first organized and then coded as a response to a specific question. Some responses were assigned more than one code, where appropriate. Subsequently, data were analyzed across the organized codes (i.e., in response to the questions posed) to look for patterns or themes in the coding. Raw data, analysis, and coding were shared and discussed with a second analyst (ST); adjustments to coding and analysis were made by consensus. A summary of the analyzed results were presented to all 33 workshop participants, including graduate students, an invited discussant, and several researchers who had not been part of the survey.

For reporting purposes, all possible identifiers of respondents were removed from the survey texts. Quotations are denoted by quotation marks and reported anonymously by respondent number. Ethical commitments to anonymity and confidentiality, while limiting description of individuals, also provides the conditions in which we can generally assume the truthfulness of respondents. Ethical approval was granted by the University of Ottawa Research Ethics Board.

## Results

A total of 23 of 24 individuals who accepted an initial invitation to attend the workshop responded to the survey. Of the 23 respondents, 3 were men and 20 were women. The estimated ages of respondents ranged from the late-20s to late-60s. Respondents came from a variety of academic, clinical, health policy and other experiential backgrounds and included university-affiliated and independent researchers, research coordinators and health-related professionals, some of whom were affiliated with non-governmental health organizations. Systematic reviews were used by respondents as sources of evidence for academic work, clinical practice, and/or policy development. Of the 23 respondents, 14 had experience with systematic reviews either as members of teams who conduct reviews or as contributors or advisors concerning review content or methods. Most were members of Cochrane and Campbell Collaborations’ methods and review groups and several were affiliated with other research organizations conducting reviews. Nine of the 23 respondents indicated a primary expertise in the area of sex/gender analysis. The respondents’ combined experience with systematic reviews and/or sex/gender was extensive and they formed a multi-disciplinary group with diverse academic backgrounds, approaches, and discourses.

Respondents provided rich and detailed answers to the questions, placing particular emphasis on the identification of challenges and thorny issues. No respondents offered examples of how they addressed these issues in their work. All respondents identified expected outcomes from the workshop. A summary of the results is provided in the following sections.

### Challenges and thorny issues

Analysis of the data revealed a number of common themes among the perceived challenges to integrating considerations of sex/gender in systematic reviews. These have been organized into four main interrelated categories: 1) conceptual challenges; 2) methodological challenges; 3) challenges related to availability and quality of data; and 4) practical and policy challenges.

#### Conceptual challenges

Clarifying the concepts of ‘sex and gender’ and putting concepts into practice in systematic reviews were identified as significant challenges. Respondents noted the tendency for sex to be used as a proxy for gender and that the terms sex and gender were used interchangeably:

“*One tricky thing has been the lack of clarity around whether it is gender or sex or both being reported in primary studies and in reviews. This makes us have to use the ever-ambiguous ‘and/or’ much of the time*” (Respondent #6).

“*Some reviewers (and other clinical researchers) may still be unclear about the concepts of ‘sex’, ‘gender’, ‘equity’, ‘race/ethnicity’, etc. and how to incorporate these*” (Respondent #21).

Others pointed to the complexity of the concepts of sex and gender, indicating that they are not single variables, but interrelated biological and social processes that in many ways have yet to be understood. Respondents stressed the need for communication between sex/gender experts and systematic reviewers to provide clarity and guidance on capturing the nuanced and intersecting nature of sex/gender particularly within the context of systematic reviews.

Beyond the definitional challenges, respondents called attention to broader conceptual challenges that often manifest within interdisciplinary projects. Specifically, respondents raised concerns about the construction of knowledge and ‘what counts’ as evidence and the challenges these present to incorporating sex/gender analysis into systematic reviews. The absence of sex/gender considerations within systematic reviews was traced back to what influences how knowledge is produced:

“*The biggest challenges are much more fundamental and have to do with the way that we arrive at decisions as to what is important for us to study, why it is important for us to study, and how we determine the way to study and ultimately produce evidence*” (Respondent #3).

“*The emergence of an evidence-based medicine approach has altered…our understanding of how to identify credible evidence and how to synthesize that evidence to inform medical practices as well as policies and programs… This approach to evidence, knowledge and policy-making has profound effects on who is authorized to provide credible accounts, what counts as credible evidence, and what counts as credible synthesis and translation*” (Respondent #16).

The assumptions about ‘credible’ evidence were seen to have particular implications for the inclusion of sex/gender analysis in systematic reviews because sex-disaggregated data and sex/gender differences and similarities are often not taken into account in the research design, conduct, data analysis, and reporting of randomized controlled trials that form the basis of most reviews. In studies that include both men and women, conclusions are often presented as applicable to all ‘subjects’. This is, in part, related to a history of excluding women in adequate numbers from many clinical trials and the assumption that findings from studies of men could be extrapolated to women [48]. As respondents noted:

“*Over time, I realised that women were being excluded from the development of evidence in a variety of ways… (and this had implications for) who were scientists, who defined the questions, who were subjects in research, what conditions were studied…*” (Respondent #11).

“[My] *concern is a lack of examination of sex/gender and other diversity within systematic reviews. There are important differences but does the current methodology hide those differences or fail to examine them…* (Respondent #14).

Dealing with diversity and heterogeneity of populations was a significant methodological challenge described by respondents as detailed below.

#### Methodological challenges

Methodological issues generated the largest amount of commentary. Some respondents expressed concerns about a lack of transparency in clinical trial design, outcomes, and reporting current practices which have implications for the quality of systematic reviews:

“*There is a lack of transparency about clinical trial design, including a priori outcomes and analyses, or poor quality protocols. There is insufficient reporting of methodologic information in current trial registries…*” (Respondent #21).

The quality of reporting in primary studies has a cascading effect on reviews and on the analyses that can be completed:

“*There are many perplexing issues with regards to conduct of systematic reviews. The most profound is quality of reporting. The quality of reporting directly leads to the comprised validity of systematic reviews, as well as contributing to the assessment of clinical heterogeneity which is a complex issue often overlooked by reviewers.*” (Respondent #10).

In particular, respondents noted that sex and gender and other health determinants are often neglected in the single studies that form the basis of reviews. They identified major challenges to both primary studies and systematic reviews:

“*…determining ways of measuring equity and bias, challenges of being able to capture complex social processes in what we mean by incorporating sex/gender.*” (Respondent #5).

“*…* [the] *question to ask is both related to quality of the research being reported and sorting out when, if, or how sex and gender matter.*” (Respondent #11).

“*There needs to be more work undertaken to explore how to identify and synthesize evidence on interventions to address structural determinants of health/health equity.*” (Respondent #16).

An approach suggested to address these challenges is through use of a range of data sources and a variety of research designs:

“*I’m wondering about the methodology and quality of the reviews that incorporate these different research designs and how we can use all the literature, not just the quantitative…to ensure a comprehensive overview of the literature, including grey literature, when we are making context-sensitive, evidence-based decisions*.” (Respondent #14).

Respondents noted a lack of available tools, checklists, and/or outlines for conducting sex/gender analysis within primary studies and systematic reviews. Tools and checklists are practical aids that incorporate recognition and understanding of what sex/gender can mean when applied into practice, and were seen as essential for those new to the concepts of sex/gender. It was recognized, that “*tools are still in development and lagging behind the theoretical arguments for inclusion of these issues*” (Respondent #9). While tools are essential, caution was expressed about relying too heavily on a tool to conduct complex analyses:

“*I struggle with the fact that I have not been able – and I am reluctant to anyway – turn sex- and gender-based analysis (or any other form of equity analysis) into a set checklist of questions and techniques. To date, my experiences have taught me that these analyses are more fluid and emergent than predictable, and depend on the history of research and evidence in the field, the framing of the question, the sources of data available, whether sex disaggregated data are available, how researchers operationalize the two concepts and the context of the question*.” (Respondent #13).

Moving from the analytical framework of sex/gender analysis towards appropriate and robust methods to address these concepts in systematic reviews raised additional challenges. Respondents warned about pitfalls when developing appropriate methods to integrate sex/gender in systematic reviews. In particular, they recognized that subgroup analyses are an important tool for investigating heterogeneity and examining results for men and women separately, but such analyses raise important technical and interpretive challenges:

“*Heterogeneous outcomes and small studies make robust subgroup analysis difficult to complete.*” (Respondent #3).

“*Few primary studies pre-specify subgroup analyses or acknowledge the limitations of analyses as hypothesis-generating. Over-interpretation of results (emphasis on the subgroup analysis rather than the primary outcome) and subsequent claims also present challenges when the emphasis is on subgroup analysis rather than the primary outcome, and give rise to potential for publication bias, and spurious (not to mention some notorious…) subgroup claims in the literature.*” (Respondent #21).

On the other hand, respondents considered it important to address the implications of potential sex/gender-based differences and equity issues in relation to health conditions and interventions:

“*Reviewers may not recognize that even small differences in the magnitude of treatment effect could translate into meaningful differences to consider in risk-benefit ratios.*” (Respondent #21).

Respondents acknowledged that methodological challenges, such as quality of data reporting and appropriate subgroup analyses, were not unique to sex/gender considerations but were of general concern to systematic reviewers. However, they identified a specific need for dialogue and guidance on the application of sex/gender analysis to systematic review methods:

“[There is a] *need for appropriate guidance and training for both trialists and systematic reviewers and for leadership in methods, and examples of applying these methods*.” (Respondent #17).

#### Challenges related to availability and quality of data

Linked to the methodological issues were challenges related to the availability and quality of data needed to conduct a robust review: these challenges became particularly marked when matters of sex/gender were considered. Many commented on the lack of data about women, lack of sex-disaggregated data, and/or inconsistencies in reporting such data:

“[There is a] *lack of sex-disaggregated data in published studies. Lack of transparency in published studies, for example, cases where some outcomes are reported for both men and women and others are not, without explanation….* [I was] *working on a review that examined a health issue specifically among women. It was challenging to find data on women to conduct the review. This stimulated questions around quality, the questions researchers ask and the answers that result from these very specific questions.*” (Respondent #3).

“*Original studies do not include the kind of disaggregated data that might allow secondary sex/gender analyses to be carried out*.” (Respondent #12).

The absence of evidence or the use of suboptimal data were regarded as issues of data quality that affect the conduct of systematic reviews generally. These were seen to stem from “*…lack of transparency, access to all the studies, all measured outcomes in studies and to the original data that might allow additional questions to be answered*” (Respondent #12). Respondents also pointed to the potential for serious consequences from the absence, omission or other shortcomings in data gathering, analysis, and reporting, such as failure to include data about adverse events.

“*A ‘thorny’ issue that deserves special mention is how to find, adequately assess, and incorporate harms data into intervention systematic reviews. This is a consistent problem due to poor quality reporting and/or measurement, suppression of data or delays in publication, short duration of follow-up, and a lack of representation of potentially vulnerable populations for whom the intervention is ultimately targeted.*” (Respondent #21).

In particular, if questions regarding sex and gender were not asked, either when conducting single trials or systematic reviews, then the data would not be available to answer questions about possible differences in treatment effects for women or men.

“*I think there is an assumption by some, that if the treatment works, why wouldn't it work for everybody? And for those convinced of the importance, many will consider it a great effort to try to obtain this additional data from the authors of the original studies*.” (Respondent #19).

In essence, the non-availability and/or non-reporting of data created an iterative cycle in which questions about sex/gender were repeatedly left out. Respondents aptly summarized this issue:

“*Questions not asked and therefore not answered.*” (Respondent #3).

“*…the questions that are asked are often driven by the data that are available* [so that] *there is some resistance to asking sex/gender/diversity questions.*” (Respondent #13).

These challenges underscore the importance of systematic reviews in driving reporting standards for primary studies. Emerging guidance on the use of sex/gender analysis in systematic reviews encourages authors to report what is known and not known about effectiveness of the intervention reviewed [49].

#### Practical and policy challenges

In addition to the difficulties of obtaining sex-disaggregated data, and a lack of tools, respondents identified other practical challenges to integrating sex/gender in systematic reviews in health. These included an absence of ‘model’ reviews to illustrate how sex/gender analysis could be carried out:

“*There are still relatively few examples of solid Cochrane reviews that incorporate a consistent approach to sex and gender analysis and equity analysis.*” (Respondent #20).

Other practical challenges dealt with the realities of working in a research intensive environment with competing demands and expanding workloads. Respondents cited experiences of resistance or ‘push back’ to incorporating considerations of sex, gender, and equity in systematic reviews “*…from authors and review groups who feel overwhelmed with the workload already demanded of them*” (Respondent #9). Respondents also discussed realities of publishing, noting “[there is] *difficulty finding journals that think this sort of research has merit*” (Respondent #7). In order to address these challenges, respondents saw the need for researchers, reviewers and editors to be aware of the substantive and growing body of health evidence addressing sex/gender similarities and differences in relation to health outcomes. A critical mass of evidence was considered integral to support the merit of such studies.

Respondents also identified several policy challenges that were system level and generally fell outside the scope of systematic review practice. Relating to earlier challenges about the availability of data, respondents described a need for policies that would mandate transparency in research and data collection, calling for increased transparency and more complete reporting of methodological information in current trial registries. These issues have important implications for the quality of systematic reviews.

“[There is] *a lack of binding guidance on the part of regulatory agencies and funders of clinical trials to collect the data in a pre-specified manner, and to make the data publicly available*.” (Respondent #21).

The types of decisions required to address these challenges are typically made and funded by higher levels of government or quasi-governmental bodies that implement government directions into policy. However, systematic reviewers working at the ‘coalface’ of reported study data have an important role to play in advocating for such policy changes.

### Proposed outcomes

While respondents described the challenges and thorny issues in detail, no one answered the question, “*How have you attempted to address these?*” However, respondents did propose a range of outcomes they hoped would emerge from the workshop. These proposed outcomes indicate how some of the identified challenges could be approached and eventually addressed. Proposed outcomes included developing methods guidelines and/or a series of methods papers to provide a framework and practical ways to integrate sex/gender analysis in systematic reviews and in peer review; refining current tools and checklists for the design and appraisal of reviews and/or developing new tools; and establishing cross-disciplinary collaborations through a ‘community of practice’. Respondents sought ways to determine the “*threshold of important differences*” of sex/gender (Respondent #13), the influence of sex/gender “*on the summary effects in a systematic review*” (Respondent #1), and “*the key ideas to help increase the sex/gender relevance of reviews…the minimum to consider to maximize the impact*” (Respondent #8). Through joint efforts, collaborators could share resources, conduct research, including model reviews, and provide educational fora, training and outreach to biomedical and social scientists, practitioners, peer reviewers, journal editors, funders and policy makers interested in the quality and applicability of health evidence. Collaboration might also result in “*a strategy to encourage funding agencies and regulatory agencies to require the collection of key data related to issues of sex/gender, equity, and bias in publicly funded trials, full disclosure of study results, and the publication of protocols*” (Respondent #21). Many of these suggestions laid the basis for initiatives discussed below.

## Discussion

Systematic review methodology provides a framework and structure for the collection, analysis, synthesis, and reporting of data, and notably in the case of Cochrane Reviews, strict protocols and methodological approaches that are regularly reviewed and updated [1]. However, despite considerable research demonstrating that sex- and gender-related processes often matter to health outcomes and an increasing number of institutional policies supporting sex/gender analysis in health research and reporting, these issues remain largely unknown or disregarded. This is the case for many areas of medical and clinical research, health technology assessment, and in conducting systematic reviews [7,50]. Asking a multidisciplinary group of individuals who conduct and/or use systematic reviews to identify what they considered to be major challenges to addressing sex/gender analysis in reviews proved invaluable to scope out key conceptual, methodological, practical, and policy issues.

In particular, respondents recognized that integrating sex/gender analysis in systematic reviews is an important step forward to answer the question “*to whom does the evidence apply and to whom might it not apply?*” in order to make sound clinical and policy decisions. They sought greater clarity in defining the concepts of sex and gender and guidance in operationalizing these concepts in the context of systematic reviews. Requests for clarity and guidance were not surprising given that these concepts have been re-defined since the 1970s by Ann Oakley, Nancy Krieger and other researchers and theoreticians, to transcend narrow assumptions of biological determinism in relation to women’s health, and that international research bodies, health organizations, and academic researchers continue to develop analytic frameworks to address sex/gender in more nuanced ways [12,51-53]. As some respondents suggested, this involves seeing sex and gender as more than discrete variables or part of a checklist of items to be ‘controlled for’, but as dynamic and entangled biological and social processes that shape human health and cross-cut other health determinants.

It was also clear from responses to the survey questions that respondents, a majority of whom had expertise in systematic review methodology, recognized the many limitations to the quality of evidence in primary studies, systematic reviews, and meta-analyses. These perspectives were evident in comments about poor quality protocols; the lack of transparency in clinical trial design; inadequate reporting of outcomes, including adverse events; in the challenges of conducting sub-group analyses; and in addressing the many forms of methods bias in appraisal of evidence. As Ioannidis and others have shown, seemingly rigorous methods have considerable strengths, “*but can also lead to wrong or misleading answers*” [54], p. 169]. For example, the outcomes of meta-analyses on the same topic may differ widely due to many factors, including that those conducting/sponsoring the meta-analyses may choose different data sources, search strategies, inclusion/exclusion criteria for eligible populations, and have different, sometimes conflicting interests that may influence how results are presented and interpreted [54].

Systematic reviewers who choose to include considerations of sex/gender and equity in their protocols are often limited by the absence of relevant data in primary research studies. Decisions taken early in the research process have implications for the subsequent synthesis of evidence. As some respondents noted, asking questions about the potential relevance of sex/gender-related processes to the research question or intervention at the protocol stage of systematic review design can help, for example, to clarify baseline from effect differences and pre-empt other gaps and omissions with regard to sex/gender.

Importantly, there is an increased interest within the Cochrane Collaboration and within the larger systematic review community as to how knowledge is produced, appraised and translated into practice, and in the theory and logic behind interventions [36,55-59]. Several groups are engaged in work to increase the quality and applicability of systematic reviews and in particular, to address issues of health equity [6,60]. Current guidelines, including the Cochrane and Campbell Health Equity checklist [61], and the Equity Extension of PRISMA (Preferred Reporting Items for Systematic Reviews and Meta-Analyses) [62], are examples of tools beginning to incorporate consideration of sex/gender. There is also increased interest in addressing context in reviews, for example, by incorporating qualitative evidence about who may or may not be affected by particular health interventions, in what ways, and why, including relevant cultural and historical conditions [63,64]. Attention to context can broaden the scope and methods of reviews to better integrate sex/gender analysis, and considerations of equity.

## Conclusions

The results of this survey identified challenges and ‘thorny’ issues associated with sex/gender in systematic reviews and emphasized that methods to integrate sex/gender analysis in reviews are still in the early stages of development. To begin to address these challenges, workshop participants suggested a number of priority items and collaborative initiatives, including the development of resources, such as ‘briefing notes’ and methods papers, to guide reviewers in the design and appraisal of systematic reviews. As part of such guidance, reviewers are encouraged, at a minimum, to disaggregate data by sex; conduct subgroup analyses, when possible, or indicate why analyses could not be performed; and address the question: To whom does this evidence apply? Reporting what is known and not known about sex/gender transparently highlights gaps in available data, contributes to future research questions, and reinforces awareness about the need for improved reporting practices about sex/gender in primary studies.

The many challenges identified in the survey may also help to explain why existing policies that encourage and/or mandate the integration of sex/gender analysis in health research are not consistently implemented. Such policies and guidelines are necessary to raise awareness of sex/gender considerations, redress past exclusions of women, minorities and others from clinical trials and improve the quality of health evidence: they require political will, direction and monitoring to ensure implementation. Based on responses to the survey and subsequent workshop discussions about the conceptual, methodological, practical, and policy challenges of integrating sex/gender analysis in systematic reviews, we suggest that the implementation of existing sex/gender policies and guidelines should be supported and reinforced through opportunities for training, collaborative projects, and cross-disciplinary dialogue among a range of stakeholders, including trial sponsors, researchers, systematic reviewers, policy makers, journal editors, research ethics boards and peer review bodies.

Spurred by the challenges and priorities identified at the workshop, a community of practice has emerged among those with expertise and interest in sex/gender analysis, health equity, and systematic review methodology to integrate sex/gender analysis into the practice of systematic reviews. Participants are working together to enhance the quality and applicability of systematic reviews, with the goals of improving health outcomes and health equity for all persons.

## Endnotes

^a^See: http://equity.cochrane.org/sex-and-gender-analysis.

^b^“Combining Forces to Improve Systematic Reviews: Gender, Equity and Bias”, funded by the Canadian Institutes of Health Research (Grant#109018).

^c^NVivo qualitative data analysis software Version 8 2008. Doncaster, Australia: QSR International Pty Ltd. [http://www.qsrinternational.com].

## Competing interests

The authors declare that they have no competing interests.

## Authors’ contributions

VR conceived and developed the survey, analyzed the data, and drafted and revised the article. ST conceived and developed the survey, analyzed the data, and drafted and revised the article. MD conceived and developed the survey, and drafted and revised the article. MB conceived and developed the survey, and helped to revise the article. All authors read and approved the final manuscript.
